# Galactomannan Testing and the Incidence of Invasive Pulmonary Aspergillosis: A 10-Year Nationwide Population-Based Study in Taiwan

**DOI:** 10.1371/journal.pone.0149964

**Published:** 2016-02-22

**Authors:** Kuo-Shao Sun, Ching-Fang Tsai, Solomon Chih-Cheng Chen, Yih-Yuan Chen, Wan-Chun Huang

**Affiliations:** 1 Division of Pulmonary and Critical Care Medicine, Department of Internal Medicine, Ditmanson Medical Foundation Chia-Yi Christian Hospital, Chiayi City, Taiwan; 2 Chung-Jen Junior College of Nursing, Health Sciences and Management, Chiayi City, Taiwan; 3 Department of Medical Research, Ditmanson Medical Foundation Chia-Yi Christian Hospital, Chiayi City, Taiwan; 4 Department of Pediatrics, School of Medicine, Taipei Medical University, Taipei, City, Taiwan; 5 Department of Internal Medicine, Ditmanson Medical Foundation Chia-Yi Christian Hospital, Chiayi City, Taiwan; Lee Kong Chian School of Medicine, SINGAPORE

## Abstract

**Background:**

The clinical impact of the galactomannan (GM) test for the diagnosis of invasive pulmonary aspergillosis (IPA) is controversial. Our study evaluated the incidence and trends of IPA and GM testing in patients with aspergillus infections.

**Methods:**

We conducted a nationwide inpatient population study using the Taiwan National Health Insurance Research Database. A total of 346 IPA (62.14% male) patients from the years 2002 to 2011 were identified for inclusion in the study.

**Results:**

The average incidence of IPA was 1.51 per million person-years. Over the study period, we observed an increasing trend from 0.94 to 2.06 per million person-years (P < 0.0001). We observed male predominance in IPA incidence (M/F: 1.85/1.15). Both males and females showed significantly increasing trends of IPA incidence over time (0.87 to 4.55 and 0.36 to 2.07 per million person-years for the males and females, respectively). GM testing for IPA significantly increased from 2002 to 2011, and the GM test was utilized more frequently for males than females. The increase in the incidence of IPA might be positively associated with the increase in GM testing over the past decade.

**Conclusion:**

The incidence rates of both IPA and GM testing have increased over time. GM testing is recommended for the early diagnosis of patients with suspected aspergillosis.

## Introduction

Invasive aspergillosis infections (IAIs) result in high mortality. The reported mortality rates range from 34% to 64.4% [1,2,3]. The majority of IAIs have pulmonary involvement, which may be isolated (61.5~94%) [4,5] or collectively involved with multiple organs (92.9~100%) [1,3,4,5]. The most important risk factor for invasive pulmonary aspergillosis (IPA) is prolonged granulocytopenia (e.g., in HIV or hematological malignancy patients or stem cell transplant recipients). However, in recent years, the epidemiology of IPAs has expanded. New risk factors are emerging, such as structural lung disease [6], chronic obstructive lung disease [7,8], autoimmune disease [9], the abuse of broad spectrum antibiotics, increased severity and complexity of disease status in the intensive care unit and evolving antifungal resistance [10].

IPA can be very challenging to diagnose because both the clinical presentation and radiographic images are often non-specific. A definitive diagnosis from a sputum culture is time consuming, and invasive procedures, such as bronchiolar lavage or tissue biopsy, are not always possible due to the patient’s existing clinical condition, such as thrombocytopenia from an underlying hematological disease.

In 2002, the galactomannan (GM) test was approved by the United States Food and Drug Association (US FDA) for use with serum samples to diagnose *Aspergillus* infections. The following year, Taiwan officials also approved the use of the GM test, and the laboratory fee became covered by national insurance. GM is a polysaccharide cell wall component that is released by *Aspergillus* species during active cell replication. Therefore, a positive GM result can indicate a true infection rather than contamination from airway commensal microbiota. According to a meta-analysis, the efficacy of the GM test in cases of proven IAI had an overall sensitivity and specificity of 71% and 89%, respectively [11]. In addition, higher titer levels correlated with superior test specificity. In some patients, the GM antigen can be detected before the presence of clinical symptoms. Early GM detection might lead to early treatment for susceptible patients prior to disease progression. Therefore, in addition to traditional radiographic images and cultures, the GM test might facilitate clinical decision-making. However, the GM test sensitivity can be lower in patients receiving antifungal treatment [12], and false-positive results have been observed in patients receiving piperacillin-tazobactam [13]. Therefore, despite the inclusion of the GM test in the diagnosis of invasive fungal infections by the European Organization for Research and Treatment of Cancer/Invasive Fungal Infections Cooperative Group and the National Institute of Allergy and Infectious Diseases Mycoses Study Group (EORTC/MSG) in 2008 [14], its clinical impact remains controversial.

Previous studies on the incidence of IPA have been conducted at medical institutions specializing in onco-hematological diseases or organ transplantation. The reported incidence of IPA varies [1,3,4]. To date, no population-based study has been performed to determine the disease incidence on a national scale. Therefore, we determined the incidence and trends of IPA in Taiwan over the past 10 years based on the national database. We also determined the potential influence of the GM test on IPA diagnoses.

## Materials and Methods

We performed a population-based study based on data obtained from the Taiwan National Health Insurance Research Database (NHIRD). Taiwan’s National Health Insurance (NHI) program was launched in March 1995 and provides compulsory medical service to all citizens with a current coverage rate exceeding 99% of the total population. NHIRD is a large-scale research database that consists of comprehensive claim records including information on patients’ characteristics, such as gender and birth date, and clinical information, such as the dates of clinical visits and admissions, medication prescription records and up to five discharge diagnoses. The diagnosis codes are based on the International Classification of Diseases, Ninth Revision, Clinical Modification (ICD-9-CM). NHI has a specific laboratory code for the *Aspergillus* antibody and antigen test. We used this laboratory code to identify the utilization of the test during the study period. The patients’ identification numbers were scrambled before data release; therefore, each subject remained anonymous. This study was approved by the Institutional Review Board of the Ditmanson Medical Foundation, Chia-Yi Christian Hospital, Taiwan (IRB number: 104071).

## Study Subjects and Definitions

We enrolled hospitalized patients who were diagnosed with IPA (ICD-9-CM codes 484.6) between 2002 and 2011. To improve the accuracy of the IPA diagnosis, we only selected patients who had received at least 3 days of antifungal therapy, such as voriconazole, posaconazole, micafungin, itraconazole, caspofungin, anidulafungin or amphotericin B during their hospitalization. Thus, we excluded patients who had empirically received antifungal therapy but subsequently received an alternative diagnosis. The demographic characteristics, resource utilizations, and geographic regions of the patients were also studied. Age at the first diagnosis was categorized into four groups: 0–18, 19–49, 50–64, and over 65 years old. We separated the study period into two five year periods to evaluate changes in the incidence rate.

## Statistical Analyses

We calculated the incidence rate of IPA stratified by sex, age groups, and time periods by calendar years (2002–2011). The incidence rate per one million person-years was calculated by dividing the number of patients with IPA by the number of the total population in the same year, which was obtained from the Department of Statistics in the Ministry of the Interior of Executive Yuan in Taiwan. We recorded the total number of patients in which the GM test was performed. The total number of GM tests was plotted according to the annual incidence rates to depict trends during the study period. A Poisson regression analysis was used to test for different temporal trends regarding incidence rates. Statistical analyses were performed using the SPSS software, Version 21 of the SPSS System for Windows (version 21.0; IBM Corporation, Somers, NY, USA). A two-tailed P-value below 0.05 was considered significant.

## Results

### Demographics

A total of 450 patients with a discharge diagnosis of IPA were identified. Among these patients, 346 had received at least 3 days of intravenous antifungal therapy and were included in our analysis. The demographic characteristics are listed in Table 1. There were 215 males and 131 females in this study. Of these patients, 90.6% of males and 87.8% of females were adults >18 years old (P = 0.0312). The male group had a shorter length-of-stay (LOS) and lower medical cost compared to the female group. However, there were no other significant differences regarding gender (e.g., use of intubation or mechanical ventilation, intensive care unit (ICU) admission, or LOS in the ICU).

**Table 1:** Demographic characteristics, clinical features, and resource utilizations of patients with invasive aspergillosis pneumonia.

Variables-Number (%)	Total (N = 346)	Male (N = 215)	Female (N = 131)	P value
**Age, years**				
**0–18**	32 (9.3)	16 (7.4)	16 (12.2)	0.0312
**19–49**	106 (30.6)	69 (32.1)	37 (28.2)	
**50–64**	99 (28.6)	53 (24.7)	46 (35.1)	
**65+**	109 (31.5)	77 (35.8)	32 (24.4)	
**Mean ± SD**	51.9 ± 21.1	53.6 ± 21.3	49.1 ± 20.7	0.0537
**Intubation and MV use**	121 (35.0)	77 (35.8)	44 (33.6)	0.6736
**ICU admission**	129 (37.3)	80 (37.2)	49 (37.4)	0.9709
**Length of stay, days- Median (IQR)**				
**Overall**	36 (21–57)	31 (20–51)	46 (28–64)	0.0001
**In ICU admission**	11 (5–23)	11 (5–23)	12 (4–19)	0.9632
**Medical cost, US dollars- Median (IQR)**	15641 (7405–29411)	14028 (6310–26013)	20246 (9998–33750)	0.0006

SD: standard deviation. IQR: inter-quartile range. MV: mechanical ventilator

### Incidence of IPA and GM testing

**Fig 1:**
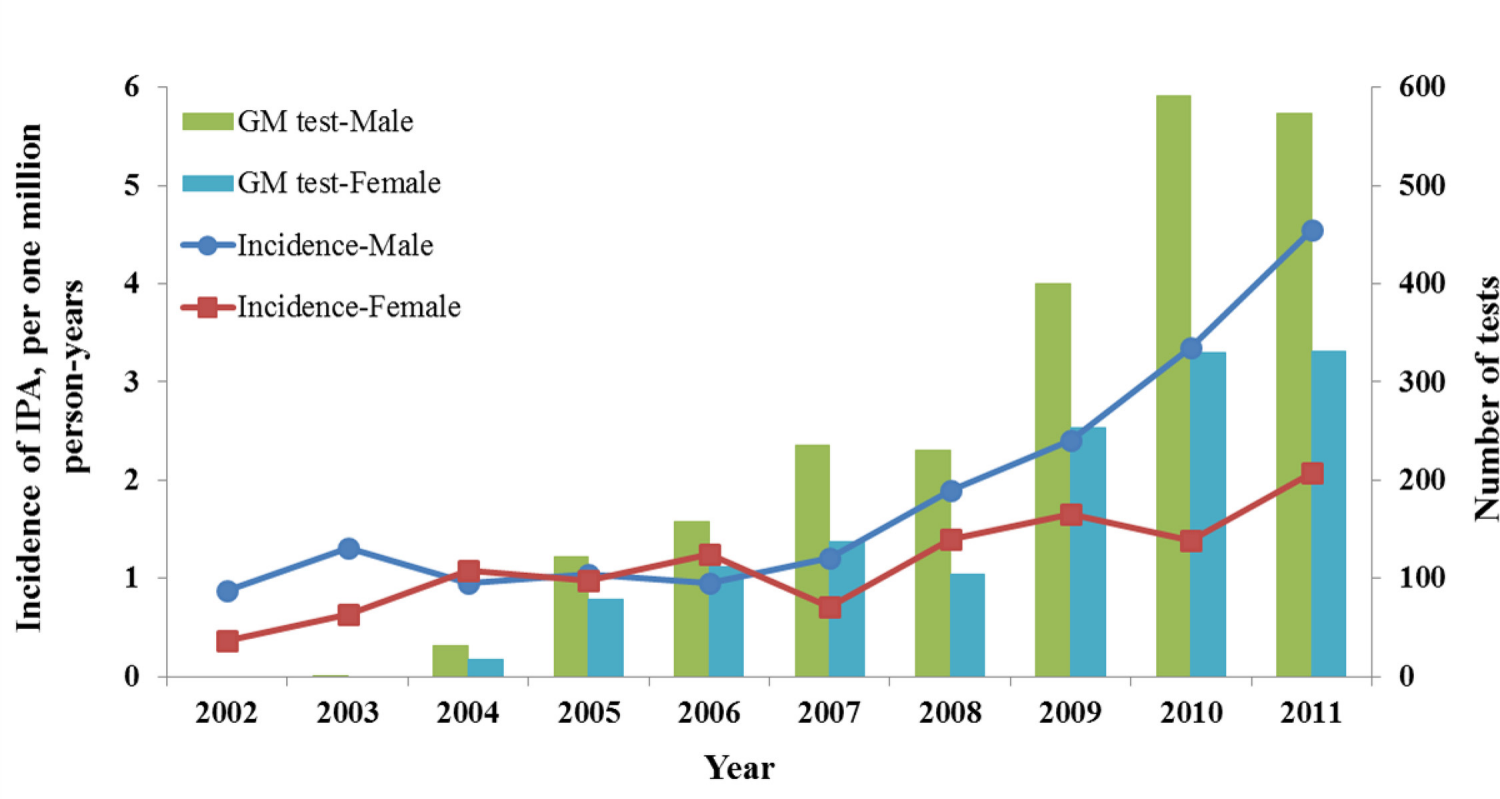
Correlation of the incidence of invasive pulmonary aspergillosis in different sex with galactomannan test utilization rate over time.

The overall incidence of IPA was 1.51 per one million person-years during the study period. The incidence of IPA in males and females was 1.85 and 1.15 per one million person-years, respectively. In Fig 1, an increasing incidence trend is observed for both males and females between 2002 and 2011 (from 0.87 to 4.55 (P < 0.0001) and 0.36 to 2.07 (P < 0.0001) per one million person-years for males and females, respectively). The number of GM tests performed for male patients increased from 0 in 2002 to 573 in 2011, and the number of GM tests performed for female patients increased from 0 in 2002 to 331 in 2011. The GM test was applied more frequently in males than females, and the increasing trend was similar to the incidence trend from 2002 to 2011.

### Incidence trend increased with age

**Fig 2:**
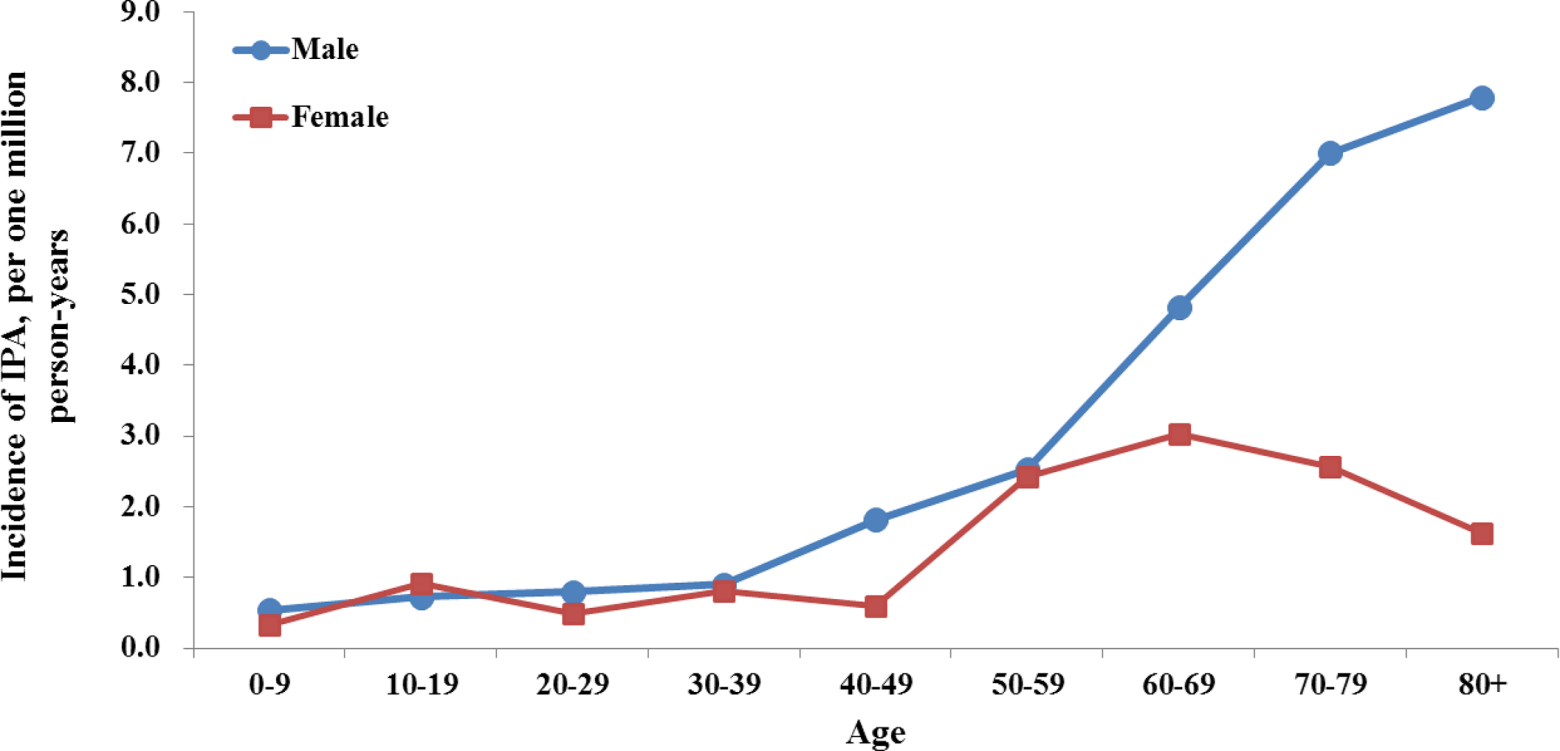
Incidence trend of invasive pulmonary aspergillosis in male and female among different age group.

Fig 2 shows that the incidence of IPA increased with age for both males and females. Among patients over 60 years of age, the incidence was higher among males than females and rapidly increased with age among males.

### Incidence change stratified by different time period

We observed that the incidence of IPA slowly increased during the 2002–2006 period and rapidly increased during the 2007–2011 period (Fig 1). We calculated the incidence of IPA for the 2002–2006 and 2007–2011 periods (Table 2). The overall incidence was higher in the 2007–2011 period than the 2002–2006 period (0.94 vs. 2.07 per one million person-years, P < 0.0001). The significantly higher incidence in 2007–2011 period was also observed when the patients were stratified into age groups and sexes (except for the age group 50–64 years, P = 0.0520).

**Table 2:** Incidence rate (per one million person-years) by time period, stratified by age and sex.

Incidence	2002–2006	2007–2011	P value
**Overall**	0.94	2.07	< 0.0001
**Sex**			
**Female**	0.86	1.45	0.0042
**Male**	1.02	2.68	< 0.0001
**Age, y**			
**0–18**	0.35	0.87	0.0181
**19–49**	0.57	1.28	0.0001
**50–64**	2.06	3.11	0.0520
**65+**	2.78	6.47	< 0.0001

## Discussion

Although the GM test has been available over the past ten years, the real clinical impact of the GM test has not been carefully validated on a national scale. The epidemiological evidence has been lacking to support the belief that the GM test is a surrogate marker for the diagnosis of IPA. This study assessed the IPA epidemiology in Taiwan over a 10 year period based on a nationwide representative database and illustrates a positive correlation between IPA incidence rate and GM test utilization. This could be a strong epidemiological evidence to confirm the use of GM test and its relationship to IPA incidence. We think the inclusion of GM test for the diagnosis of “probable IA” in the revised guideline and increased availability are major reasons for more use of GM test over the past years.

This study nationally assessed the IPA epidemiology in Taiwan over a 10 year period. The incidence rate of IPA remained low during the 2002–2006 period and rose rapidly after 2007. The incidence rate of IPA correlated well with the use of the GM test, which suggests an association between these factors.

Invasive aspergillosis affected more males than females, which has been demonstrated in some previous studies (Table 3). Two studies from the United States reported that the percentage of male patients was 55.9% and 58.9% [2,15]. Another study performed by Lortholary et al. (393 patients) also showed a male predominance (62%) [3]. Perkhofer et al.[1] and Hsiue et al.[4] also found similar results. In this study, 62.1% of the IPA patients were male, which is in agreement with previous reports [1,4]. The reason(s) for the gender difference is unknown. However, because IPA develops more often in patients with specific diseases, the gender difference might be due to the epidemiological features of those underlying diseases. For example, hematological malignancies, the most widely recognized condition associated with IPA, affects significantly more male than female patients [16]. In addition, IPA can occur among patients who have HIV infections, and men have a higher risk of progression to AIDS [17]. For these patients, the possibility of invasive *Aspergillus* infections is higher due to compromised immunity. Another epidemiological study of invasive aspergillosis among intensive care unit patients showed that among 563 patients who acquired invasive aspergillosis, 31% had chronic obstructive pulmonary disorder (COPD), which is also a male-predominant disease [18].

**Table 3:** Comparison of large epidemiological studies on invasive aspergillosis.

Author	Country	Study period	Patient source	Incidence	Male
**Kim et al.**	United States	2000 ~ 2006	Insurance company data-bank	-	55.9%
**Steinbach et al.**	United States	2004 ~ 2008	25 Medical centers	-	58.9%
**Lortholary et al.**	France	2005 ~ 2007	12 Teaching hospitals	271/1000,000 admission	62%
**Perkhofer et al.**	Australia	2007 ~ 2008	12 Major transplant or oncology centers	23,6/1000,000 hospital inhabitants	58%
**Hsiue et al.**	Taiwan	2000 ~ 2009	Single medical center	-	58%

Currently, there is no literature available regarding the age-specific IPA incidence rate. In our cohort, the age-specific incidence rate was lowest among children less than 10 years old, and the rate increased with age. For males, the increased incidence rate with age may be a result of the epidemiological trends of the underlying comorbidities. In one cohort of patients with invasive aspergillosis, 22.9% had acute myeloid leukemia (AML) and 21.4% had undergone allogeneic hematopoietic stem cell transplantation (HSCT) [3]. AML generally occurs in adults, and its incidence increases with age. According to Marr et al., older age was associated with an increased risk of invasive aspergillosis among 1,682 patients who received their first allogeneic HSCT [19]. This study, which covered all ages of the population, provided information regarding the age distribution of IPA.

Fig 1 shows that the number of GM tests increased gradually during the study period, and was found to be correlated with the number of IPA cases. This result corroborates a previous single-institute study that found a significant increase in the diagnosis of IAI in patients with hematological malignancies after GM testing (11 cases in 2011 versus 2 cases in 2007) [20]. The researchers postulated that this increase in diagnosis was due to a classification shift from “possible” to “probable” IA. We suggest that the initial low incidence rate was caused by a lack of awareness and difficulty of diagnosis in 2007. Previous researches showed that 60 to 75% of invasive aspergillosis was diagnosed post mortem [21,22] and the vast majority had pulmonary involvement, which suggested that the diagnostic and therapeutic strategies were suboptimal and needed to be improved. GM testing had become an elementary component of the diagnosis of “probable” IA in 2008, according to the EORTC/MSG consensus. The present study revealed a sudden increase in IPA cases since 2008, which provides strong epidemiological evidence to support the use of GM testing for improving the diagnosis of IPA. Although the GM test has been in use for greater than 10 years and was included in the criteria for “probable” IA by the EORTC/MSG consensus in 2008, its clinical impact remains unknown. In previous studies, the performance of the GM test varied significantly using different clinical scenarios and cut-off values [23]. Indeed, one study demonstrated that the serum GM test rarely impacted clinical decision-making and indicated that it might not be cost-effective [24].

There are several limitations should be addressed in this study. First, the validity of the diagnoses was limited. Patients with aspergillosis are most effectively diagnosed on the basis of the EORCT-MSG 2008 criteria. However, we were unable to apply the EORCT/MSG 2008 criteria to define the IPA cases because information on clinical symptoms and culture results are not available in administrative claims data. We acknowledged this weakness and attempted to overcome it. Following Kim et al. [15], we used strict inclusion criteria, i.e. hospitalized patients receiving at least 3 days of intravenous anti-fungal medications, which we believe covers the patients most likely to have IPA. Second, only those hospitalized patients were included. Thus, patients with mild cases of IPA who had not been admitted to the hospital were not studied. Nevertheless, due to the nature of this disease, most patients diagnosed with IPA would be hospitalized. Therefore, the number of missing cases due to mild cases should be too small to influence the general epidemiological trends. Third, the current analysis did not include cases diagnosed post mortem. This might influence the incidence rate of IPA of this study. However, the fact that autopsies are rarely performed in Taiwan would minimize such effect on the reported rates. Fourth, this study just focused on basic demographic data such as gender, age and time of occurrence. Additional factors like intubation and mechanical ventilation, ICU admission, length of stay and medical cost are also important variables worthy of discussion. However, they are beyond the scope of the current study and may be studied in the future.

## Conclusions

In conclusion, this study provided a nationwide assessment of IPA epidemiology over a 10 year period. We observed similar trends for the incidence rate of IPA and the rate of GM test administration. The incidence of IPA increased significantly during the study period, and the improved diagnostic rate may be due to the utilization of the GM test.

## References

[1] Perkhofer S, Lass-Florl C, Hell M, Russ G, Krause R, Honigl M, et al The Nationwide Austrian Aspergillus Registry: a prospective data collection on epidemiology, therapy and outcome of invasive mould infections in immunocompromised and/or immunosuppressed patients. Int J Antimicrob Agents. 2010; 36: 531–536. 10.1016/j.ijantimicag.2010.08.010 20947312

[2] Steinbach WJ, Marr KA, Anaissie EJ, Azie N, Quan SP, Meier-Kriesche HU, et al Clinical epidemiology of 960 patients with invasive aspergillosis from the PATH Alliance registry. J Infect. 2012;65: 453–464. 10.1016/j.jinf.2012.08.003 22898389

[3] Lortholary O, Gangneux JP, Sitbon K, Lebeau B, de Monbrison, Le Strat Y, et al Epidemiological trends in invasive aspergillosis in France: the SAIF network (2005–2007). Clin Microbiol Infect. 2011;17: 1882–1889. 10.1111/j.1469-0691.2011.03548.x 21668573

[4] Hsiue HC, Wu TH, Chang TC, Hsiue YC, Huang YT, Lee PI, et al Culture-positive invasive aspergillosis in a medical center in Taiwan, 2000–2009. Eur J Clin Microbiol Infect Dis. 2012;31: 1319–1326. 10.1007/s10096-011-1445-1 21997774

[5] Montagna M, Lovero G, Coretti C, Martinelli D, Delia M, De Giglio O, et al SIMIFF study: Italian fungal registry of mold infections in hematological and non-hematological patients. Infection. 2014;42: 141–151. 10.1007/s15010-013-0539-3 24150958 PMC3906525

[6] Segal BH. Aspergillosis. N Engl J Med. 2009; 360: 1870–1884. 10.1056/NEJMra0808853 19403905

[7] Guinea J, Torres-Narbona M, Gijon P, Munoz P, Pozo F, Pelaez T, et al Pulmonary aspergillosis in patients with chronic obstructive pulmonary disease: incidence, risk factors, and outcome. Clin Microbiol Infect. 2010;16: 870–877. 10.1111/j.1469-0691.2009.03015.x 19906275

[8] Bulpa P, Dive A, Sibille Y. Invasive pulmonary aspergillosis in patients with chronic obstructive pulmonary disease. Eur Respir J. 2007;30: 782–800. 17906086 10.1183/09031936.00062206

[9] Chen HS, Tsai WP, Leu HS, Ho HH, Liou LB. Invasive fungal infection in systemic lupus erythematosus: an analysis of 15 cases and a literature review. Rheumatology (Oxford). 2007;46: 539–544. 17043051 10.1093/rheumatology/kel343

[10] Vandewoude KH, Blot SI, Depuydt P, Benoit D, Temmerman W, Colardyn F, et al Clinical relevance of Aspergillus isolation from respiratory tract samples in critically ill patients. Crit Care. 2006;10: R31 16507158 10.1186/cc4823 PMC1550813

[11] Pfeiffer CD, Fine JP, Safdar N. Diagnosis of invasive aspergillosis using a galactomannan assay: a meta-analysis. Clin Infect Dis. 2006;42: 1417–1427. 16619154 10.1086/503427

[12] Marr KA, Laverdiere M, Gugel A, Leisenring W. Antifungal therapy decreases sensitivity of the Aspergillus galactomannan enzyme immunoassay. Clin Infect Dis. 2005; 40: 1762–1769. 15909264 10.1086/429921

[13] Walsh TJ, Shoham S, Petraitiene R, Sein T, Schaufele R, Kelaher A, et al Detection of galactomannan antigenemia in patients receiving piperacillin-tazobactam and correlations between in vitro, in vivo, and clinical properties of the drug-antigen interaction. J Clin Microbiol. 2004;42: 4744–4748. 15472335 10.1128/JCM.42.10.4744-4748.2004 PMC522332

[14] De Pauw B, Walsh TJ, Donnelly JP, Stevens DA, Edwards JE, Calandra T, et al Revised definitions of invasive fungal disease from the European Organization for Research and Treatment of Cancer/Invasive Fungal Infections Cooperative Group and the National Institute of Allergy and Infectious Diseases Mycoses Study Group (EORTC/MSG) Consensus Group. Clin Infect Dis. 2008;46: 1813–1821. 10.1086/588660 18462102 PMC2671227

[15] Kim A, Nicolau DP, Kuti JL. Hospital costs and outcomes among intravenous antifungal therapies for patients with invasive aspergillosis in the United States. Mycoses. 2011;54: e301–312. 10.1111/j.1439-0507.2010.01903.x 20557463

[16] Dorak MT, Karpuzoglu E. Gender differences in cancer susceptibility: an inadequately addressed issue. Front Genet. 2012;3: 268 10.3389/fgene.2012.00268 23226157 PMC3508426

[17] Jarrin I, Geskus R, Bhaskaran K, Prins M, Perez-Hoyos S, Muga R, et al Gender differences in HIV progression to AIDS and death in industrialized countries: slower disease progression following HIV seroconversion in women. Am J Epidemiol. 2008;168: 532–540. 10.1093/aje/kwn179 18663213

[18] Taccone FS, Van den Abeele AM, Bulpa P, Misset B, Meersseman W, Cardoso T, et al Epidemiology of invasive aspergillosis in critically ill patients: clinical presentation, underlying conditions, and outcomes. Crit Care. 2015;19: 7 10.1186/s13054-014-0722-7 25928694 PMC4344741

[19] Marr KA, Carter RA, Boeckh M, Martin P, Corey L. Invasive aspergillosis in allogeneic stem cell transplant recipients: changes in epidemiology and risk factors. Blood. 2002;100: 4358–4366. 12393425 10.1182/blood-2002-05-1496

[20] Hoenigl M, Salzer HJ, Raggam RB, Valentin T, Rohn A, Woelfler A, et al Impact of galactomannan testing on the prevalence of invasive aspergillosis in patients with hematological malignancies. Med Mycol. 2012;50: 266–269. 10.3109/13693786.2011.603102 21905944

[21] Chamilos G, Luna M, Lewis RE, Bodey GP, Chemaly R, Tarrand JJ, et al Invasive fungal infections in patients with hematologic malignancies in a tertiary care cancer center: an autopsy study over a 15-year period (1989–2003). Haematologica. 2006;91: 986–989. 16757415

[22] Sinko J, Csomor J, Nikolova R, Lueff S, Krivan G, Remenyi P, et al Invasive fungal disease in allogeneic hematopoietic stem cell transplant recipients: an autopsy driven survey. Transpl Infect Dis. 2008;10: 106–109. 17605727 10.1111/j.1399-3062.2007.00264.x

[23] Arvanitis M, Mylonakis E. Diagnosis of invasive aspergillosis: recent developments and ongoing challenges. Eur J Clin Invest. 2015;45: 646–652. 10.1111/eci.12448 25851301

[24] Prasad P, Fishman JA. Impact and cost of the serum galactomannan assay at a tertiary care facility. Transplantation. 2014;98: 773–780. 10.1097/TP.0000000000000131 24825524

