# Correction: Galactomannan Testing and the Incidence of Invasive Pulmonary Aspergillosis: A 10-Year Nationwide Population-Based Study in Taiwan

**DOI:** 10.1371/journal.pone.0156566

**Published:** 2016-06-01

**Authors:** Kuo-Shao Sun, Ching-Fang Tsai, Solomon Chih-Cheng Chen, Yih-Yuan Chen, Wan-Chun Huang

There is an error within Table 3. The country in Row 4 is incorrectly labeled as ‘Australia’. The correct country should be: Austria. Please see the corrected Table 3 here.

**Table 3:** Comparison of large epidemiological studies on invasive aspergillosis.

Author	Country	Study period	Patient source	Incidence	Gender (M)
Kim et al.	United States	2000 ~ 2006	Insurance company data-bank	-	55.9%
Steinbachet al.	United States	2004 ~ 2008	25 medical centers	-	58.9%
Lortholary et al.	France	2005 ~ 2007	12 teaching hospitals	271/1000,000per admission	62%
Perkhoferet al.	Austria	2007 ~ 2008	12 major transplant or oncology centers	23,6/1000,000 hospital inhabitants	58%
Hsiue et al.	Taiwan	2000 ~ 2009	Single medical center	-	58%
